# Spinal Cord Stimulation for Non-Reconstructable Chronic Limb-Threatening Ischemia: A Real-World, Multidisciplinary, Single-Center Experience

**DOI:** 10.3390/jcm15051760

**Published:** 2026-02-26

**Authors:** Naoufel Ouerchefani, Edward Goldberg, Pascal Desgranges

**Affiliations:** 1 Neurosurgery Department, Foch University Hospital, 92150 Suresnes, France; 2 Boston Scientific Neuromodulation, Valencia, CA 91355, USA; 3 Vascular Surgery Department, Henri Mondor University Hospital, 94000 Creteil, France

**Keywords:** claudication, ischemic pain, limb amputation, peripheral artery disease, spinal cord stimulation

## Abstract

**Background/Objectives**: Chronic limb-threatening ischemia (CLTI) is a severe form of peripheral artery disease characterized by ischemic rest pain or ulcer necrosis. In Europe, spinal cord stimulation (SCS) can be offered to CLTI patients with chronic pain to improve mobility and prolong limb preservation. We evaluated the long-term, real-world outcomes of SCS therapy in patients with CLTI. **Methods**: In this observational study, medical chart review data from consecutive CLTI patients treated with SCS were analyzed. **Results**: Fifty-three patients (56.6% Fontaine Stage III, 39.6% Fontaine Stage IV, 3.8% Fontaine Stage IIb) had a single-stage SCS implant procedure between 2013 and 2022. Two years after SCS therapy activation, claudication pain intensity had significantly improved; the overall numerical rating scale pain score decreased from 9.4 ± 0.9 at baseline to 3.7 ± 3.2 (*p* < 0.0001). In addition, walking distance increased by more than 350 m (from 70 ± 87 to 429 ± 320 m, *p* < 0.0001), and pre-existing skin lesions stabilized in ten patients (63%). The probability of limb survival in Fontaine’s stage IIb/III and Fontaine’s stage IV patients at 12 months was 90% and 70%, respectively (log-rank *p*-value = 0.04). Finally, significant associations were found between the occurrence of an amputation after SCS and Fontaine Stage (*p* = 0.01), active smoking (*p* = 0.02), hypertension (*p* = 0.04), and prior minor amputation (*p* = 0.02). No major complications were reported. **Conclusions**: Our real-world experience suggests that SCS for CLTI patients provides significant and durable improvements in ischemic pain and functional outcomes. SCS may also help reduce the natural risk of major amputation, especially when implemented at early CLTI stages.

## 1. Introduction

Lower extremity peripheral artery disease (PAD) affects approximately 230 million people globally [1,2]. PAD is characterized by chronic impairment of arterial perfusion, most commonly involving multilevel atherosclerotic disease. While early stages of PAD are usually asymptomatic, disease progression leads to exertional ischemia and the development of intermittent vascular claudication as skeletal muscle oxygen demand exceeds arterial supply. Approximately 11% of patients with PAD develop chronic limb-threatening ischemia (CLTI), the most advanced stage of the disease, characterized by ischemic rest pain or tissue loss [3]. Patients with CLTI present with multiple serious comorbidities and have high mortality rates (20% at 6 months; up to 50% at 5 years)—particularly in patients with wounds ineligible for vascular reconstruction. Major amputations with CLTI are frequent, ranging from 10 to 40% at 1 year, leading to reduced quality of life [4].

Revascularization plays a central role in managing symptomatic lower extremity PAD by improving perfusion, thereby enhancing mobility, reducing ischemic pain, and improving quality of life. In advanced clinical stages such as CLTI, management requires a multidisciplinary approach that includes optimizing comorbidities, wound care, and vascular reconstruction [5]. In 2019, the Global Vascular Guidelines introduced a framework for CLTI management, emphasizing individualized risk stratification to guide revascularization strategies [4]. Tools such as GLASS (limb anatomic stage) and WIfl (wound, ischemia, and infection stage [6]) facilitate assessment of anatomic complexity, ischemic severity, and limb threat to identify patients most likely to benefit from vascular reconstruction. Despite advances in revascularization and standardized decision frameworks, a subset of patients with CLTI derive limited or no durable benefit from surgical intervention, and repeated revascularization attempts may accelerate clinical deterioration. Inoperability in CLTI patients is typically determined based on the anatomical distribution of the disease and a comparative assessment of benefits versus surgical risks. For patients with non-reconstructable CLTI and disabling ischemic rest pain, alternative strategies are required to address symptoms and preserve quality of life.

Spinal cord stimulation (SCS) is an established therapy for the management of chronic intractable neuropathic pain of the trunk and/or limbs, where application of electrical energy generated by an implantable pulse generator (IPG) via leads implanted in the epidural space at specific spinal levels stimulates nerves in the spinal cord. SCS was originally grounded in the gate control theory of pain proposed by Melzack and Wall in 1965 [7], which postulated that modulation of the dorsal horn signaling could attenuate nociceptive transmission to the brain. Conventional SCS produces a “tingling” sensation (paresthesia) that helps to reduce chronic neuropathic pain. The electrical current can be shaped to optimize selective stimulation of the fibers innervating the painful area based on its dermatomal topography, thereby reducing the pain sensation [8,9,10]. Newer SCS programming options have been developed over the last 10 years, offering paresthesia-free and combination therapies [11,12,13,14,15,16,17].

Since its first use in 1976 for patients with peripheral vascular disease [18], the effectiveness of SCS in patients with late-stage PAD (Fontaine Stage III or IV) has been extensively evaluated. Multiple clinical studies, including randomized controlled trials, have demonstrated clinically meaningful benefits of SCS in CLTI patients, including reductions in ischemic rest pain, improvements in mobility and quality of life, enhanced wound healing, and increased limb salvage rates [19,20,21,22,23,24,25,26,27,28,29].

Beyond pain relief, SCS has been associated with modulation of sympathetic tone and microvascular perfusion—mechanisms of particular relevance in ischemic limb disease. Several studies have further reported improvements in microcirculatory parameters, including capillary flow, capillary density, and foot skin temperature, suggesting a potential effect of SCS on limb perfusion [22,30,31]. While these effects could partially be attributed to increased physical activity, additional research was conducted to better characterize SCS mechanisms of action in peripheral ischemic pain. This postulated that SCS could improve limb microcirculation via antidromic activation of sensory fibers, thereby inducing the release of vasodilators [32,33]. These findings prompted clinicians to use additional patient selection criteria, such as superficial tissue perfusion as measured by TcPO2 (transcutaneous partial pressure of oxygen), as a predictive factor of success, especially for limb salvage [34,35,36,37,38,39].

Despite these encouraging findings, the role of SCS in the contemporary management of CLTI remains incompletely defined. Much of the available evidence originates from studies conducted prior to the widespread adoption of modern endovascular techniques and before the introduction of current neuromodulation paradigms, including paresthesia-free and combination stimulation approaches. In addition, heterogeneity in patient selection, outcome measures, and definitions of treatment success has limited the generalizability of historical data to current clinical practice.

Nevertheless, multiple professional societies have acknowledged SCS as a potential non-revascularization therapeutic option for carefully selected patients with advanced PAD who are unsuitable for durable surgical or endovascular intervention. An expert consensus statement published in the Journal of the American College of Cardiology, and the 2017 European Society of Cardiology guidelines on the diagnosis and treatment of PAD, recognize SCS as an adjunctive therapy for patients with CLTI and refractory ischemic breast pain when revascularization is not feasible or has failed [40,41]. Similarly, recommendations from the European Society for Vascular Surgery and the 2019 Global Vascular Guidelines for the Management of Chronic Limb-Threatening Ischemia emphasize individualized risk stratification and acknowledge SCS as a non-revascularization therapy that may reduce ischemic pain and amputation risk in selected patients [4].

Despite inclusion in international guidelines, these recommendations consistently highlight the limited availability of contemporary real-world data evaluating functional outcomes, limb salvage, and quality of life following SCS implantation in patients with CLTI. In particular, there remains a need to better characterize outcomes in patients treated with modern stimulation paradigms within routine clinical practice.

Accordingly, further evaluation of SCS patients with advanced PAD, particularly those with non-reconstructable CLTI and persistent ischemic symptoms, remains warranted. The present study reports a retrospective analysis of a consecutive case series of patients with CLTI treated with SCS in a real-world, multidisciplinary clinical practice. The primary objectives were to assess the impact of SCS on ischemic pain, functional mobility, and limb salvage, and to contextualize these findings within the evolving framework of contemporary CLTI management.

## 2. Materials and Methods

Our single-center, retrospective analysis of medical chart data from a consecutive case series of CLTI patients in France is part of an ongoing, international, observational, chart review study that is characterizing real-world clinical outcomes of SCS for the treatment of chronic pain using de-identified data from the sites’ medical records (ClinicalTrials.gov, NCT01550575). The study includes multiple independent cohorts to evaluate outcomes in different subgroups. The cohort in our study comprises patients with chronic pain due to CLTI who received SCS, with data obtained from de-identified patient records in a consecutive case series. All data were collected by site personnel, as per standard practice, and entered directly into an electronic data collection system without sponsor involvement.

The study site obtained Ethics Committee approval (Local Ethics Committee CNIL, protocol code 2214468 v 0, 19 July 2019). All patients provided informed consent, as required by local regulatory requirements, and the study was conducted in accordance with Good Clinical Practice (ISO 14155) guidelines [42] and the Declaration of Helsinki.

### 2.1. Study Setting and Participants

All adult patients (≥18 years) treated with SCS at the study center were eligible for inclusion, provided that device implantation was consistent with the approved directions for use and local regulatory requirements. Patient selection was conducted through close collaboration between the vascular surgery and neurosurgery departments. Patients with CLTI were initially evaluated by the vascular surgery team, which determined suitability for surgical or endovascular revascularization based on anatomical considerations, comorbidities, and expected procedural risk-benefit. Patients deemed non-reconstructable or unlikely to derive durable benefit from revascularization were referred to the neurosurgery department for further evaluation. Final candidacy for SCS implantation was determined on an individual basis in accordance with standard clinical practice at the study site. Implantation decisions incorporated CLTI-specific clinical considerations routinely applied at the center, including ischemic rest pain refractory to conservative management, functional impairment, and overall prognosis. Patients with extensive pre-existing ulcers (>3 cm^2^ [34]), active ulcer infection, or an estimated life expectancy ≤ 1 year due to comorbid conditions were not implanted, consistent with local standard-of-care practices.

For the present retrospective analysis, consecutive patients with chronic lower limb ischemia pain due to non-reconstructable CLTI who underwent SCS implantation and for whom baseline and follow-up clinical assessments were available were included. Following implantation, stimulation modalities and programming strategies were selected in accordance with routine clinical practice at the discretion of the treating physician.

### 2.2. Implementation of SCS Therapy

The SCS leads were placed using standard techniques and according to the implanting physician’s preferences. When the SCS system was implanted, all patients underwent a single-stage procedure with a surgical lead and a non-rechargeable IPG. Programming of the SCS device was conducted by the implanting physician and the pain management team, according to their preference and the capabilities of the implanted device. Besides standard rate (i.e., paresthesia-based SCS), newer sub-perception waveforms were used either at the initial postoperative stage or later during the follow-up visits. Reprogramming sessions were organized in accordance with standard practice, based on observed outcomes and patient feedback during follow-up visits.

### 2.3. Outcome Measures

All patients were assessed prior to SCS implantation and during follow-up visits, organized by the study site according to their standard practice. Demographic information, pain location, medical, procedural, and surgical history were all recorded. The Fontaine scale was used to document the PAD stage and clinical presentation of patients at baseline (Stage I, asymptomatic; Stage II, mild claudication pain in limb (Stage IIa, claudication at a distance > 200 m, Stage IIb, claudication at a distance < 200 m); Stage III, rest pain, mostly in the feet; Stage IV, necrosis and/or gangrene of the limb). The numerical rating scale (NRS; from 0 (no pain) to 10 (worst pain)) was used to evaluate pain intensity (score ≤ 3, mild pain; 4–6, moderate pain; ≥7, severe pain). Where documented, walking distance, revascularization and amputation procedures, and ulcer presence, size, and evolution were analyzed. Adverse events (AEs) were collected and documented by the site in patients’ medical records, per standard practice; however, the protocol did not include documentation of safety variables in study case report forms.

The real-world, retrospective study design meant that data collection and analyses were based on patient clinical assessments recorded in pre-existing documentation, as performed by the site in accordance with its standard follow-up practice. As a result, the number of observations for each datapoint may differ and fluctuate over time. Results presented in this study include data for up to 2 years after SCS implantation.

### 2.4. Statistical Analysis

In this predefined analysis, a Kolmogorov–Smirnov test was used to confirm the normality of the NRS score change. Mean values and standard deviations were calculated for baseline demographic data and pain scores. A paired *t*-test with a two-sided 0.05 significance level was used to assess whether the average decrease in baseline pain score was greater than 0. For continuous variables, results are presented as mean ± standard deviation. Results are presented as frequency (percentage) for categorical variables. Survival methods were used to analyze the cumulative rate of major amputations over time. Log-rank tests were used to compare Kaplan–Meier curves. Fisher’s exact test was used to assess the association of baseline characteristics with amputation events. The analyses were generated using SAS/STAT software, Version 9.4 of the SAS System for Windows. Copyright © 2023 SAS Institute Inc. SAS and all other SAS Institute Inc. product or service names are registered trademarks or trademarks of SAS Institute Inc., Cary, NC, USA.

## 3. Results

### 3.1. Patient Characteristics

In total, 53 CLTI patients (mean age 64.4 ± 13.7 years, 64% male) were analyzed (Table 1). All patients were de novo SCS cases, implanted between July 2012 and October 2023. Over the study period, SCS systems from three manufacturers were used (Medtronic, Minneapolis, MN, USA: n = 34; Boston Scientific, Valencia, CA, USA: n = 12; Abbott Medical, Plano, TX, USA: n = 7) with 16-contact surgical paddle leads. Non-rechargeable neurostimulators were used in 49 patients, and rechargeable devices were implanted in four patients as replacements for non-rechargeable devices when their batteries became depleted. Using documented programming information from 53 patients, paresthesia-based SCS therapy and sub-perception SCS waveforms were used in 62% and 38% of cases, respectively.

At baseline, patients reported severe pain (mean NRS 9.4 ± 0.9, n = 47) and markedly limited walking capacity (mean 69.5 ± 86.9 m, n = 43). Most subjects were either in Fontaine Stage III (rest pain) or IV (trophic lesions), and CLTI comorbidities were frequent, including arterial hypertension, diabetes, and hyperlipidemia. Fourteen of the 21 Fontaine Stage IV patients had documented information about their skin lesions: 10 had ulcers on their toe(s) and 4 on other parts of their feet, with an average ulcer size of 2.74 cm^2^. A total of 38 vascular reconstruction procedures had been performed and documented for 25 patients before SCS implantation, eight of whom had undergone multiple revascularization procedures. The most common revascularization procedures were superficial femoral artery and femoropopliteal bypasses. Eight patients had an amputation prior to SCS, five minor ones involving toe(s), two below the knee, and one above the knee.

### 3.2. Pain Relief and Walking Distance After SCS Therapy

**Figure 1:**
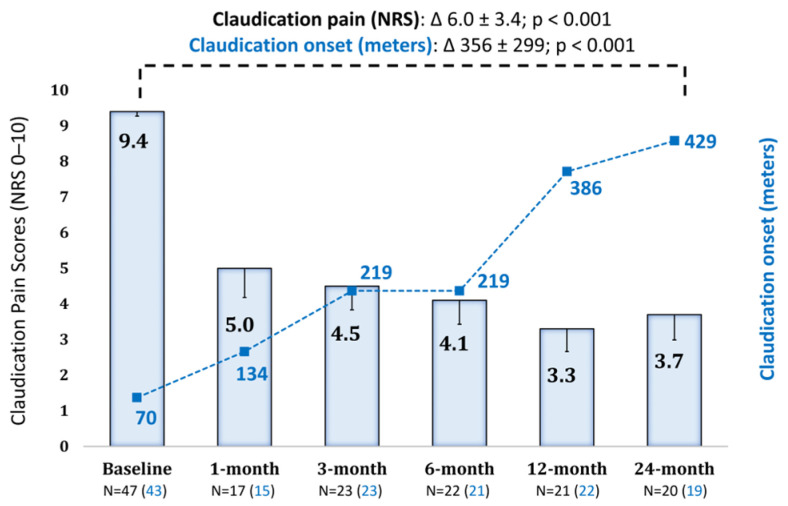
Effects of SCS on pain intensity, walking distance, and limb survival. Evolution of pain (NRS 0–10) and claudication onset distance at up to 2-year follow-up after SCS implantation. Values presented as mean (standard error). NRS = numerical rating scale.

In all patients, ischemic rest pain was eliminated shortly after device activation. With regard to claudication pain, the overall NRS pain score decreased to 3.7 ± 3.2 (mean change −6.0 ± 3.4, n = 20, *p* < 0.0001) at 2 years, while patients’ walking distance significantly improved by an average of 356 ± 299 m (*p* = 0.0002) (Figure 1).

### 3.3. Skin Lesions and Limb Salvage

Among 16 Fontaine Stage IV patients with documented ulcers at 2 years, 10 (63%) showed stable or improved healing following SCS (average ulcer size 2.6 cm^2^).

**Figure 2:**
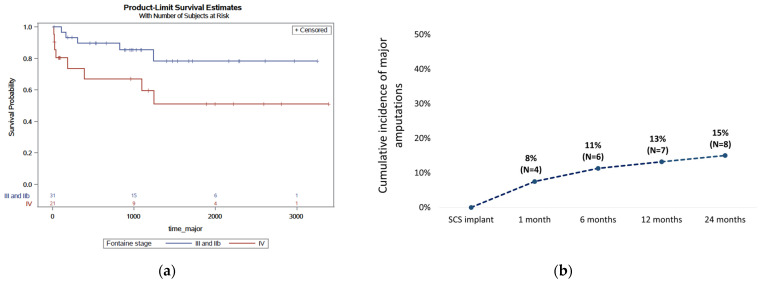
Limb survival after spinal cord stimulation. (**a**) Kaplan–Meier limb survival rates for major amputations in Fontaine Stage IIb/III versus Fontaine Stage IV patients; (**b**) major amputations after spinal cord stimulation therapy in patients with non-reconstructable chronic limb-threatening ischemia: cumulative rate over 2 years.

The overall probability of limb survival for all patients (n = 53) was 78% at 12 months (% patients free of minor and/or major amputations). The analysis of major amputations (Figure 2a) showed that the probability of limb survival in Fontaine’s stage IIb/III (n = 31) and Fontaine’s stage IV (n = 21) patients at 12 months was 90% and 70%, respectively (log-rank *p* = 0.04).

At 2 years, 45/53 patients (85%) were free from major amputation (Figure 2b). Eight (15%) had their lower limb amputated above the ankle. These major amputations took place 574 days (around 19 months) on average after SCS implantation, and were all below-the-knee procedures. Five of these eight patients (63%) were Stage IV patients; in four of these patients, the amputations took place within 1 month after SCS implantation. Overall, five patients (24%) in Fontaine Stage IV at baseline had a major amputation within 2 years after SCS implantation. In contrast, major amputations were conducted in only 3/30 (10%) of all Fontaine Stage III patients.

Finally, significant associations were found between amputation after SCS and the following baseline factors: Fontaine Stage (*p* = 0.01), active smoking (*p* = 0.02), hypertension (*p* = 0.04), and prior minor amputation (*p* = 0.02).

### 3.4. Safety

The patients’ medical files indicated that no major complications (infection, spinal cord compression, etc.) related to SCS devices and/or single-stage SCS implant procedures were reported. Over the course of the post-implantation period, device programming was adjusted to optimize outcomes and/or address discomfort or suboptimal coverage of the pain area reported by patients.

## 4. Discussion

Our single-center, observational study provides real-world evidence to support the use of SCS as an effective therapy for patients with non-reconstructable CLTI. Our findings demonstrate significant and sustained improvements in ischemic rest pain, claudication pain, and walking distance, as well as an 85% limb preservation rate at 2-year follow-up. These results align with prior studies that have established SCS as a viable option for pain relief and limb salvage in patients with advanced PAD unsuitable for revascularization [43,44,45,46].

The marked reduction in the NRS pain score (−5.7 points) observed over 2 years reflects a clinically meaningful improvement in pain control, consistent with reports of SCS efficacy in 60–82% of PAD patients [19,20,21,22,23,24,25,26,27]. Similarly, the substantial increase in walking distance (>350 m) highlights the functional benefits of SCS, corroborating earlier observations of enhanced mobility and quality of life [44,47]. These outcomes suggest that SCS both alleviates pain and supports patients in maintaining ambulatory status—a key treatment goal in symptomatic PAD.

The mechanisms underlying the benefits of SCS in CLTI remain incompletely understood but may involve improved microvascular perfusion and vasodilation. Preclinical and clinical studies have reported increases in skin temperature, capillary flow, and TcpO2 following SCS [22,30,36], potentially enhancing tissue nutrition and ulcer stabilization, as observed in 63% of our Stage IV patients with pre-existing skin lesions.

With respect to limb salvage, we report a 15% cumulative major amputation rate at 2 years, which is notably lower than the natural history of CLTI, where amputation rates can reach 25–35% within 6–12 months without intervention [48]. This reduction is particularly striking, given that nearly 40% of our patients were in Fontaine Stage IV at baseline—a group with a historically higher risk of limb loss (up to 67.3% in Rutherford class 6 patients [48]). However, our data also revealed a stage-dependent effect: 63% of major amputations occurred in Fontaine Stage IV patients, compared to 10% in Stage III—echoing findings by Piedade et al. [45]. Their retrospective analysis of 72 CLI patients who underwent SCS implantation showed that the probability of limb survival at 12 months was markedly higher in patients with Fontaine Stage III (94%) than in those with Fontaine Stage IV (62%), with major amputation occurring later in Stage III patients. This suggests that SCS may be most effective when implemented before the appearance of severe tissue loss, potentially delaying or preventing progression to amputation in Stage III patients. Future research should also explore whether earlier intervention in claudicant patients could further improve functional outcomes, and possibly delay the need for vascular reconstruction, offering a preventive role for SCS in PAD progression.

The successful use of various SCS waveforms in our study, including paresthesia-based and sub-perception modalities, aligns with emerging evidence that newer paradigms (e.g., high-frequency or burst stimulation) may play an important role in the future evolution of SCS when used in CLTI patients [44,49]. In 2024, Kretzschmar and colleagues published a retrospective analysis of 51 patients, which supported the efficacy of SCS for improving limb salvage, pain relief, mobility, and quality of life [44]. Furthermore, a (non-statistical) waveform comparison showed that subthreshold burst stimulation and suprathreshold tonic stimulation produced similar therapeutic outcomes. Later that year, Dr. Kilchukov et al. published their investigation of subthreshold high-frequency stimulation (1 kHz) compared to low-frequency stimulation (suprathreshold tonic) on pain relief in peripheral vascular disease, in a randomized trial with 56 patients [49]. High-frequency stimulation provided better pain relief, quality of life, and functional activity in CLTI patients.

Several limitations must be acknowledged. The retrospective nature of our study induced an inherent risk of incomplete data and uneven follow-up rates. Data collected in the study were sourced from patients’ medical files and relied on pre-existing documented assessments as performed per standard of care by healthcare professionals in both vascular surgery and neurosurgery departments. Some objective vascular measures, such as TcpO2 or ankle-brachial index, were not systematically documented and could not be analyzed in our report, limiting physiological insights into the effects of SCS on microvascular perfusion. Per standard practice, all patients had been diagnosed with CLTI and deemed “non-reconstructable” per the expert judgment of the vascular surgery team. As proposed by the recent “Global Vascular Guidelines”, future research could also implement additional characterization of the patients’ condition, for example, via the WIfI classification [6]. Beyond Fontaine Stage, the use of new criteria, such as GLASS and WIfI, could improve our ability to describe the variety of CLTI clinical presentations and help identify the best candidates for SCS. While standard practices in this field typically use SCS as a last resort solution for CLTI patients and as an alternative to immediate amputation, we believe refined patient characterization could help select the right patients at the right time.

Our patients’ medical records showed that no serious adverse events (i.e., spinal cord compression, infection, …) were reported during the SCS implant procedure and over the course of follow-up visits. However, the retrospective study database did not formally enable the documentation of complications; hence, safety aspects could not be analyzed in detail. The retrospective design of our study also precluded a statistical comparison of waveform efficacy, underscoring the need for much larger cohorts and/or randomized controlled trials to evaluate the differential effects of SCS modalities on pain, claudication, and limb salvage.

Claudication outcomes (pain and walking distance) could not be evaluated in more than 20 patients for up to 2 years due to various reasons (lost to follow-up, major amputation, mortality), which may have led to misinterpretation of our results. We suggest that future real-world studies be conducted within the framework of a multicenter international registry to maximize the patient population and strengthen long-term outcome analysis across multiple domains (pain, function, limb survival, microvascular perfusion, etc.). In addition, the limited sample size and number of major amputation events in this cohort precluded adequately powered multivariable regression analyses to adjust for potential confounding factors.

Our single-center report reflects the experience and clinical practice we have developed in our institution for the management of non-reconstructable CLTI patients, and the reported results are directly related to the multidisciplinary collaboration between our vascular surgery and neurosurgery departments. Our cohort included consecutive CLTI patients with various clinical presentations, comorbidities, and prior treatments, which may have introduced heterogeneity in outcomes. Despite these constraints, the real-world setting of our study enhances its generalizability to clinical practice, where patient diversity and resource availability often shape treatment decisions. It is important to note that, despite robust evidence supporting the benefits of SCS therapy in CLTI patients, including citations in cardiovascular guidelines, access to SCS remains challenging in most countries. One reason is that health authorities offer limited, if any, coverage for the procedure in this indication. Another factor is the referral pathway and how vascular patients are identified for neuromodulation treatment. Access to SCS for CLTI patients requires strong collaboration among vascular surgeons, neurosurgeons, and anesthesiologists. Our cohort of patients could be treated with SCS through a specific reimbursement process and thanks to a strong multidisciplinary partnership that prioritizes patient care and offers access to all treatment options.

## 5. Conclusions

In this single-center, real-world cohort, SCS was associated with meaningful and sustained improvements in ischemic pain, functional mobility, and limb preservation in patients with non-reconstructable CLTI. Notably, limb salvage was achieved in 85% of patients at 2 years—a rate that compares favorably with the reported natural history of CLTI and suggests a potential attenuation of major amputation risk in this high-risk population. The observed limb preservation benefit appeared to be stage-dependent, with more favorable outcomes among patients treated at Fontaine Stage III than among those presenting at more advanced stages. Beyond clinical outcomes, our experience underscores the importance of multidisciplinary collaboration and efficient referral pathways between vascular and neurosurgical teams to facilitate early patient selection and access to neuromodulation therapy. While these results should be interpreted in light of the study’s observational design, they contribute real-world evidence supporting the role of SCS as an adjunctive non-revascularization option in carefully selected patients with CLTI.

## Figures and Tables

**Table 1:** Characteristics of patients with non-reconstructable chronic limb-threatening ischemia at baseline (N = 53).

Characteristics	Patients (N = 53)
Sex–males	34 (64.2)
Age–years	64.4 ± 13.7 (n = 51)
Fontaine Stage	
IIb	2 (3.8)
III	30 (56.6)
IV	21 (39.6)
Comorbidities:	
Arterial hypertension	44 (83.0)
Diabetes	22 (41.5)
Hyperlipidemia	18 (33.9)
Cardiopathy	11 (20.7)
Active smoker	7 (13.2)
Cardiac arrhythmia	4 (7.5)
Prior revascularization procedures: Total number of procedures	38
SFA bypass	11
Femoropopliteal bypass	5
Common femoral artery bypass	3
Other bypass	5
SFA stent	5
Thrombectomy/endarterectomy	4
Percutaneous transluminal angioplasty	3
Not specified	2
Prior amputation procedures	8 (15.1)
Overall pain intensity—NRS	9.4 ± 0.9 (n = 47)
Claudication onset—m	69.5 ± 86.9 (n = 43)
Patients with foot ulcers	14 (26.4)
Last documented follow-up (Duration after SCS implant)—years	3.5 (2.7)

Results are presented as n (%) or mean ± standard deviation. NRS = numerical rating scale; SCS = spinal cord stimulation; SFA = superficial femoral artery.

## Data Availability

The data, analytic methods, and study materials for this clinical study will be made available to other researchers in accordance with the Boston Scientific Data Sharing Policy (https://www.bostonscientific.com, accessed on 14 February 2024).

## Abbreviations

The following abbreviations are used in this manuscript:

AE(s)	Adverse event(s)
CLTI	Chronic limb-threatening ischemia
NRS	Numerical rating scale
PAD	Peripheral artery disease
PVD	Peripheral vascular disease
SCS	Spinal cord stimulation
SFA	Superficial femoral artery
TcPO2	Transcutaneous partial pressure of oxygen
WIfl	Wound, ischemia, and infection stage
